# Efficacy of second-line treatment and prognostic factors in patients with advanced malignant peritoneal mesothelioma: a retrospective study

**DOI:** 10.1186/s12885-021-08025-x

**Published:** 2021-03-20

**Authors:** Rui Kitadai, Tatsunori Shimoi, Kazuki Sudo, Emi Noguchi, Yusuke Nagata, Ryoichi Sawada, Atsuo Takashima, Narikazu Boku, Kan Yonemori

**Affiliations:** 1 Department of Breast and Medical Oncology, National Cancer Center Hospital, 5-1-1 Tsukiji, Chuo-ku, Tokyo, 104-0045 Japan; 2 Department of Gastrointestinal Medical Oncology, National Cancer Center Hospital, 5-1-1 Tsukiji, Chuo-ku, Tokyo, 104-0045 Japan; 3 Division of Gastroenterology and Hepatology, Department of Internal Medicine, The Jikei University School of Medicine, 3-19-18 Nishishinbashi, Minato-ku, Tokyo, 105-8471 Japan

**Keywords:** Malignant peritoneal mesothelioma, Second-line chemotherapy, Efficacy, Prognosis

## Abstract

**Background:**

Standard treatment for malignant peritoneal mesothelioma has not been established, and systemic chemotherapy is administered according to malignant pleural mesothelioma. We previously reported the efficacy of cisplatin plus pemetrexed as first-line chemotherapy; however, the efficacy of second-line chemotherapy remains unknown.

**Methods:**

We retrospectively evaluated patients with malignant peritoneal mesothelioma who started first-line systemic chemotherapy with platinum plus pemetrexed between March 2007 and February 2019 at the National Cancer Center Hospital. Patients who received second-line chemotherapy after failure of platinum plus pemetrexed were identified. We evaluated the efficacy of first- and second-line chemotherapy, and explored the prognostic factors. Survival outcomes were estimated using the Kaplan–Meier method, and between-group differences were compared using the log-rank test. Univariate and multivariate analyses were performed using Cox proportional hazards models.

**Results:**

A total of 54 and 26 patients received platinum plus pemetrexed as first- and second-line chemotherapy, respectively (gemcitabine in 12 patients; taxane, six; nivolumab, three; and others, five). In all patients, the median overall survival and progression-free survival after first-line chemotherapy were 16.6 and 7.3 months, respectively. Among patients who received second-line chemotherapy, the median overall survival, progression-free survival, and second-line overall survival were 16.9, 3.2, and 9.9 months, respectively. Patients who received ≥6 cycles of platinum plus pemetrexed as first-line chemotherapy had longer overall survival after second-line chemotherapy than those who did not (hazard ratio, 0.23; 95% confidence interval: 0.06–0.82; *p* = 0.02).

**Conclusions:**

Second-line chemotherapy may be an option for refractory malignant peritoneal mesothelioma, especially in patients who have completed 6 cycles of platinum plus pemetrexed as first-line chemotherapy.

**Supplementary Information:**

The online version contains supplementary material available at 10.1186/s12885-021-08025-x.

## Background

Malignant mesothelioma is a rare malignancy arising from mesothelial cells of the pleura, peritoneum, pericardium, and tunica vaginalis testis [1]. The vast majority arise from the pleura, and malignant peritoneal mesothelioma (MPeM) accounts for approximately 15–20% of all cases [2], which is the second most frequent primary site. The incidence of MPeM in industrialised countries ranges between 0.5 and 3 cases per million in men and between 0.2 and 2 cases per million in women [3]. Because of the rarity of the disease, no standard treatment has been established based on randomised controlled trials. Cytoreductive surgery and hyperthermic intraperitoneal chemotherapy have been shown to improve survival. However, not all patients with MPeM are suitable for cytoreductive surgery and hyperthermic intraperitoneal chemotherapy. Systemic chemotherapy is a reasonable option for those who do not wish to undergo surgery, as well as those with biphasic or sarcomatoid high-risk histology, extra-abdominal disease, and a poor performance status (PS) [4]. However, few studies have been conducted specifically on MPeM, and systemic chemotherapy recommended for the treatment of malignant pleural mesothelioma (MPlM) is widely used.

Chemotherapy drugs for mesothelioma are considered to be equally effective regardless of the organ involved, although there are some biological differences depending on the primary site. In a randomised phase III trial published in 2003 [5], systemic chemotherapy with pemetrexed plus cisplatin for MPlM prolonged survival, with a median survival of 12.1 months compared with 9.3 months in the cisplatin alone arm (*p* = 0.02), establishing pemetrexed plus cisplatin as the current standard of care for MPlM. The efficacy of pemetrexed plus cisplatin in patients with MPeM has been reported in two studies, with response rates of 20 and 29.8%, respectively, and a median survival of 13.1 months in one study and not reached in the other [6, 7]. In our retrospective study [8], the combination of cisplatin and pemetrexed for MPeM was effective, and the median progression-free survival (PFS) and overall survival (OS) were 7.1 and 15.4 months, respectively. Replacing cisplatin with carboplatin has been shown to result in similar treatment efficacy [7, 9]. Current data supports combination chemotherapy with pemetrexed and cisplatin/carboplatin as an option for first-line treatment. As for second-line treatment, however, the efficacy remains unknown for MPeM refractory to platinum-based chemotherapy, and no second-line treatment regimens are currently recommended.

This study was conducted as an expanded analysis of the efficacy of first-line platinum-based chemotherapy, following our previous study [8]. Furthermore, we evaluated second-line treatment and explored the prognostic factors in patients who received second-line treatment.

## Methods

### Patient selection

This retrospective study evaluated patients with MPeM who started systemic chemotherapy between March 2007 and February 2019 at the National Cancer Center Hospital, Tokyo, Japan. Patients were histologically proven to have MPeM. The diagnosis of MPeM was confirmed by at least two board-certified pathologists at our or another hospital. Five patients received workers’ compensation. Ten patients were given financial relief by the Act on Asbestos Health Damage Relief.

Patients received either cisplatin plus pemetrexed or carboplatin plus pemetrexed as first-line chemotherapy. Carboplatin (area under curve 5) and pemetrexed (500 mg/m^2^) or cisplatin (75 mg/m^2^) and pemetrexed (500 mg/m^2^) were administered intravenously on day 1 of a 21-day cycle for 6 cycles [5]. Some patients continued treatment with pemetrexed alone as maintenance therapy. Maintenance chemotherapy with pemetrexed after 6 cycles of cisplatin or carboplatin plus pemetrexed was included as first-line chemotherapy. Second-line chemotherapy using various agents was administered to some patients. Treatment was continued until documented or clinical disease progression, unacceptable toxicity, deterioration of general condition, or patient refusal to continue chemotherapy.

The study protocol was approved by the Institutional Review Board of the National Cancer Center Hospital, Tokyo, Japan (approval numbers: 2012–335 and 2017–229). Research was conducted in accordance with the Declaration of Helsinki. The requirement for informed consent was waived by the Institutional Review Board owing to the retrospective nature of the study. Patients could refuse to participate in this study by an opt-out form on the website of our institution.

### Data collection

Clinical data regarding a history of asbestos exposure, age, sex, Eastern Cooperative Oncology Group (ECOG) PS, histology, amount of ascites, metastatic sites, and the number of cycles of first-line chemotherapy were collected from medical records. The number of cycles included both platinum plus pemetrexed and maintenance pemetrexed following platinum plus pemetrexed. Clinical responses were evaluated in patients with measurable lesions, according to the Response Evaluation Criteria in Solid Tumours (version 1.1) [10]. OS was calculated from the first day of systemic chemotherapy for MPeM until death or the date of last follow-up, while PFS was defined as the period from the first day of systemic chemotherapy for MPeM until documented disease progression or death prior to disease progression. PFS and OS were estimated separately from initiating first- and second-line chemotherapy. The data cut-off date was 8th February 2020.

The study protocol was approved by the National Cancer Center Research Ethics Review Committee at the National Cancer Center Hospital, Tokyo, Japan (approval numbers: 2012–335 and 2017–229). All chemotherapies were started with the patient’s consent, and patients could refuse to participate in this retrospective study by an opt-out form on the website of our institution.

### Statistical analyses

Continuous variables were compared using the *t*-test for normally distributed data and the Mann–Whitney *U* test for non-normally distributed data, while categorical variables were compared using Fisher’s exact test. The Kaplan–Meier method was used to estimate OS and PFS, and survival curves were compared using the log-rank test. Cox proportional hazards models were used to evaluate several risk factors. We included clinically relevant covariates without missing values (age, ECOG PS, and distant organ metastasis) in a multivariate Cox proportional hazards model. All *p*-values were based on two-sided tests, with *p* < 0.05 considered statistically significant. Statistical analyses were conducted using R software version 3.6.2 (R foundation for Statistical Computing, Vienna, Austria).

## Results

### Clinical outcomes of first-line platinum-based chemotherapy

A total of 54 patients with MPeM received platinum plus pemetrexed as first-line chemotherapy; 26 of whom received second-line chemotherapy. No rare subtypes (well-differentiated papillary/deciduoid mesothelioma) were present in this cohort. The baseline characteristics at the start of first-line treatment are shown in Table 1.

**Table 1:** Baseline characteristics

Characteristic	Patients (*n* = 54)
Age (years), median (range)	63 (20–82)
Age categorisation (years), n (%)	
< 70	41 (75.9)
≥ 70	13 (24.1)
Sex, n (%)	
Male	37 (68.5)
Female	17 (31.5)
Asbestos exposure, n (%)	
Yes	15 (27.7)
No	30 (55.6)
Unknown	9 (16.7)
ECOG PS, n (%)	
0	22 (40.7)
1	30 (55.6)
2	2 (3.7)
Histology, n (%)	
Epithelioid	31 (57.4)
Sarcomatoid	4 (7.4)
Mixed	4 (7.4)
Multicystic	1 (1.9)
Unknown	14 (25.9)
Ascites at initial diagnosis, n (%)	
Yes	43 (79.6)
No	11 (20.4)
Previous surgery, n (%)	
Yes	6 (11.1)
No	48 (88.9)
Distant metastasis, n (%)^a^	
Liver	5 (9.3)
Lymph node	4 (7.4)
Lung	4 (7.4)
Pleural	1 (1.9)
Bone	2 (3.7) (One patient had bone and liver metastases)
Others	5 (9.3)
None	34 (63.0)
Measurable lesions, n (%)	
Yes	25 (46.3)
No	29 (53.7)

Abbreviations: *ECOG* Eastern Cooperative Oncology Group; *PS* performance status

^a^ Some patients had multiple metastases in multiple organs

The median age was 63 (range, 20–82) years, and 37 (63.5%) patients were men. A history of obvious asbestos exposure was observed in 15 (27.7%) patients. Twenty-two (40.7%) patients had an ECOG PS of 0; 30 (55.6%), 1; and one (3.7%), 2. The histological subtype was epithelioid in 31 (57.4%) patients; sarcomatoid, four (7.4%); mixed, four (7.4%); multicystic, one (1.9); and unknown, 14 (25.9%). Reasons for unknown histological subtypes included a diagnosis at a different hospital, a cellblock or cytological diagnosis using immunocytochemistry, and not being able to perform the classification. Forty-three (79.6%) patients had ascites at initial diagnosis of MPeM. Twenty-one (38.9%) patients had distant organ metastasis and 25 (46.3%) had measurable lesions. All patients received cisplatin plus pemetrexed as first-line chemotherapy, except one patient who received carboplatin plus pemetrexed (Table 2).

**Table 2:** Treatment lines

Treatment	Patients
First-line treatment	*n* = 54 (% in the first-line treatment)
Cisplatin plus pemetrexed	53 (98)
Carboplatin plus pemetrexed	1 (2)
Second-line treatment	*n* = 26 (% in the second-line treatment)
Gemcitabine	12 (46)
Taxane	6 (23)
Nivolumab	3 (12)
Others	5 (19)
Third- or higher-line treatment	*n* = 12

**Fig. 1:**
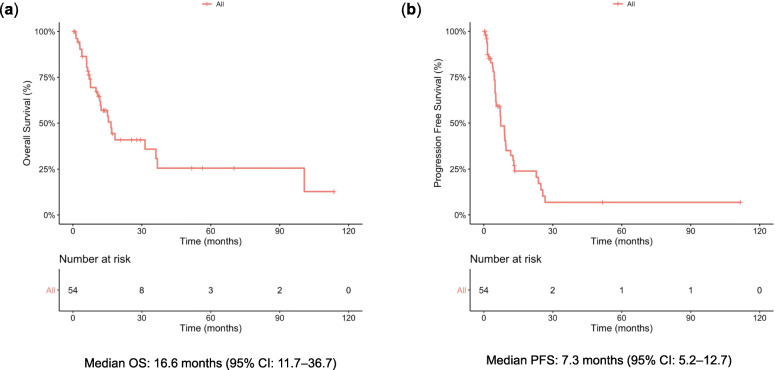
Kaplan–Meier curves for all patients. **a** OS and **b** PFS. CI, confidence interval; OS, overall survival; PFS, progression-free survival

Among patients who received first-line chemotherapy, the median OS was 16.6 (95% confidence interval [CI]: 11.7–36.7) months and the median PFS was 7.3 (95% CI: 5.2–12.7) months (Fig. 1a–b). Reasons for discontinuation of first-line chemotherapy included disease progression (including clinical disease progression; *n* = 34), deterioration of general condition (*n* = 3), patient refusal (*n* = 3), hospital transfer (*n* = 5), watchful waiting after a few cycles (*n* = 3), completion of the planned 6 cycles (*n* = 2), and others (*n* = 3). One patient was still undergoing first-line chemotherapy. Twenty-eight patients received ≥6 cycles of chemotherapy, and 26 received < 6 cycles. The overall response rate (ORR) of patients with measurable lesions was 20% (95% CI: 6.8–40.7) (see Additional file 1). Univariate analysis showed no significant association between OS and age, sex, amount of ascites, asbestos exposure, histology, ECOG PS, and distant metastasis (see Additional file 2).

### Clinical outcomes of second-line chemotherapy

Twenty-six (48%) patients received second-line chemotherapy after failure of platinum doublet chemotherapy: gemcitabine in 12 patients; taxane, six; nivolumab, three; and others, five. The patients’ background at the initiation of second-line chemotherapy was as follows: median age, 63 (range, 43–82) years; 20 (76.9%) patients were men. A history of obvious asbestos exposure was observed in eight (30.8%) patients. Eleven (42.3%) patients had an ECOG PS of 0; 14 (53.9%), 1; and one (3.8%), 2. The histological subtype was epithelioid in 14 patients (53.9%); sarcomatoid, three (11.5%); mixed, two (7.6%); and unknown, seven (27.0%). Fourteen (53.9%) patients had distant organ metastasis. Seventeen (63.4%) patients received ≥6 cycles of first-line chemotherapy (Table 3). Reasons for not receiving second-line chemotherapy, except for one patient who was still undergoing first-line chemotherapy, included a poor ECOG PS (*n* = 6), continued observation (*n* = 4), hospital transfer (*n* = 6), patient refusal (*n* = 5), and others (*n* = 6).

**Table 3:** Patient characteristics and univariate analysis of second-line overall survival after second-line chemotherapy

Characteristic		Patients (*n* = 26)	Median 2nd-line OS (95% CI), months	HR (95% CI)	*p*-value
Median age, years (range)		63 (43–82)	9.92 (4.76–NA)	–	–
Age categorisation	< 63	13	7.33 (7.23–NA)	1.34 (0.50–3.65)	0.56
	≥ 63	13	10.22 (1.91–NA)	–	–
Sex	Male	20	9.92 (4.76–NA)	0.93 (0.30–2.89)	0.9
	Female	6	7.23 (3.22–NA)	–	–
Asbestos exposure	Yes	8	7.34 (2.30–NA)	2.00 (0.68–5.87)	0.2
	No or unknown	18	10.22 (3.22–NA)	–	–
Histology	Epithelioid	14	12.55 (4.76–NA)	1.11 (0.41–3.04)	0.83
	Others	12	8.62 (1.91–NA)	–	–
ECOG PS	0–1	25	9.92 (3.22–NA)	NA	0.56
	2	1	NA (NA–NA)	–	–
Distant metastasis	Yes	14	10.22 (2.30–NA)	1.00 (0.37–2.70)	1.0
	No	12	7.33 (4.76–NA)	–	–
Cycles of platinum doublet	< 6	9	1.79 (0.66–NA)	0.23 (0.07–0.80)	0.013
	≥ 6	17	10.22 (7.23–NA)	–	–

Abbreviations: *CI* confidence interval; *HR* hazard ratio; *ECOG* Eastern Cooperative Oncology Group; *NA* not assessed; *OS* overall survival; *PS* performance status

**Fig. 2:**
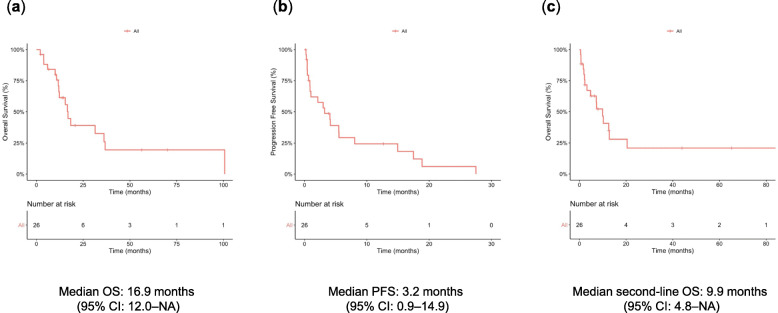
Kaplan–Meier curves for patients who received second-line treatment. **a** OS, **b** PFS, and **c** second-line OS. CI, confidence interval; NA, not assessed; OS, overall survival; PFS, progression-free survival

**Fig. 3:**
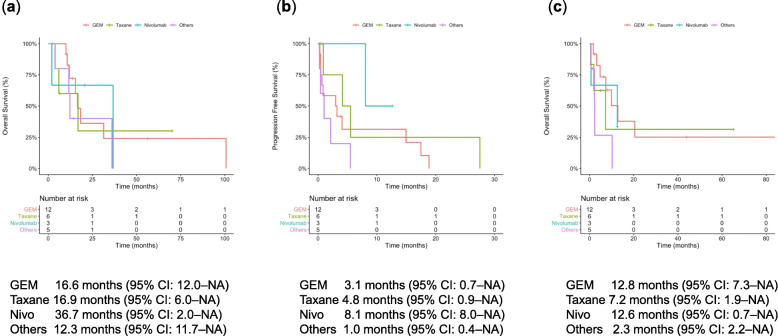
Kaplan–Meier curves according to second-line chemotherapy. **a** OS, **b** PFS, and **c** second-line OS. CI, confidence interval; GEM, gemcitabine; NA, not assessed; Nivo, nivolumab; OS, overall survival; PFS, progression-free survival

In the 26 patients who received second-line chemotherapy, the median first-line OS (time from first-line chemotherapy to death) was 16.9 (95% CI: 12.0–not assessed [NA]) months (Fig. 2a). After initiating second-line chemotherapy, the median PFS (time from second-line chemotherapy) was 3.2 (95% CI: 0.9–14.9) months (Fig. 2b), and the median second-line OS (time from second-line chemotherapy to death) was 9.9 (95% CI: 4.8–NA) months (Fig. 2c). According to the agents used in second-line chemotherapy, the median first-line OS, second-line OS, and PFS were 16.6, 12.8, and 3.1 months for gemcitabine; 16.9, 7.2, and 4.8 months for taxane; 36.7, 12.6, and 8.1 months for nivolumab; and 12.3, 2.3, and 1.0 months for others, respectively (Fig. 3a–c).

Univariate analysis showed no significant association between second-line OS and age, sex, obvious asbestos exposure, histology, ECOG PS, and distant metastasis. However, patients who received ≥6 cycles of first-line chemotherapy had longer second-line OS than those who did not (Table 3) (see Additional file 3). The multivariate analysis, which did not include ECOG PS, because only one patient had an ECOG PS of 2, also showed that ≥6 cycles of platinum plus pemetrexed as first-line chemotherapy was independently associated with longer OS (*p* = 0.02; Table 4). The median second-line OS (time from second-line chemotherapy to death) was 10.2 months in patients who completed 6 cycles of first-line chemotherapy and 1.8 months in those who did not.

**Table 4:** Multivariate analysis of second-line overall survival

Covariate	HR (95% CI)	*p-*value
Age (years), ≥ 63 (vs. < 63)	1.16 (0.41–3.27)	0.78
Cycles of platinum doublet chemotherapy, ≥ 6 (vs. < 6)	0.23 (0.06–0.82)	0.02
Distant metastasis, no (vs. yes)	0.84 (0.30–2.36)	0.74

Abbreviations: *CI* confidence interval; *HR* hazard ratio

We compared the first-line OS between patients who received second-line chemotherapy and those treated with first-line platinum doublet chemotherapy only; the median OS was 16.9 vs. 15.0 months, respectively, with no statistically significant difference (*p* = 0.99) (see Additional file 4). Second-line OS was not associated with PFS after first-line chemotherapy (*p* = 0.09). In addition, no significant association was observed between PFS after first-line chemotherapy and prognosis after second-line chemotherapy (*p* = 0.24).

Table 5 shows the adverse events. Among haematological toxicities, grade 3/4 leukopenia, neutropenia, and anaemia were observed in one (3.8%), two (7.7%), and three (11.5%) patients, respectively. Nausea, anorexia, and fatigue were among the most common non-haematological toxicities. Grade 2 rash was observed in one patient treated with nivolumab, which was considered to be an immune-related adverse event. There were no treatment-related deaths.

**Table 5:** Summary of adverse events of second-line chemotherapy

Toxicity	Any Grade, n (%)	Grade 3/4, n (%)
Haematological toxicity		
Leukopenia	8 (30.8)	1 (3.8)
Neutropenia	6 (23.0)	2 (7.7)
Anaemia	14 (53.8)	3 (11.5)
Thrombocytopenia	3 (11.5)	0 (0.0)
Non-haematological toxicity		
Nausea	7 (26.9)	0 (0.0)
Vomiting	3 (11.5)	0 (0.0)
Anorexia	7 (26.9)	0 (0.0)
Fatigue	8 (30.8)	0 (0.0)
Elevated creatinine	5 (19.2)	0 (0.0)
Diarrhoea	2 (7.7)	1 (3.8)
Constipation	5 (19.2)	0 (0.0)
Skin rash	1 (3.8)	0 (0.0)
Neurological disorder	3 (11.5)	1 (3.8)
Dysgeusia	2 (7.7)	0 (0.0)
Alopecia	3 (11.5)	0 (0.0)

## Discussion

This is the first retrospective study to investigate the efficacy of second-line chemotherapy in patients with MPeM, including monotherapy with various chemotherapy agents and immune checkpoint inhibitors. While there are a few reports on the efficacy of cisplatin plus pemetrexed as first-line chemotherapy for MPeM, the efficacy of second-line chemotherapy remains unknown.

As first-line chemotherapy for MPeM, the expanded access programme in the United States showed an ORR of 29.8% and a median OS of 13.1 months [6]. Another expanded access programme in Europe reported an ORR of 20% [7]. Several retrospective studies, including this updated analysis, have shown similar efficacy.

As for second-line chemotherapy, there are a few reports on MPlM. Several differences have been reported between MPeM and MPIM. Compared to MPIM, MPeM is more common in younger patients and in woman, and asbestos exposure is a less important risk factor [11]. The genomic profiles are also distinct, suggesting that the dysregulated pathways may vary between them [12]. However, despite the differences between MPeM and MPIM, it is assumed that the effectiveness of chemotherapy will be similar [13]. Some benefits of vinorelbine have been suggested for refractory MPlM. In a phase II trial [14], the median OS was 9.6 months in patients with previous exposure to chemotherapy. Rechallenge with pemetrexed-based therapy resulted in a median second-line OS of 13.6 months in patients who achieved disease control during first-line chemotherapy for MPlM [15]. Moreover, a longer median OS was shown in patients with MPlM who received second-line chemotherapy than in those who did not (15.3 vs. 9.8 months, respectively) [16]. In contrast, the efficacy of second-line chemotherapy in MPeM remains unknown. Gemcitabine and docetaxel have also shown efficacy with a median OS of 8 and 12.2 months, respectively, in chemo-naïve patients with MPlM [17, 18]. In this study, gemcitabine was the most commonly used regimen. Patients who received second-line chemotherapy showed similar efficacy compared to previous reports of MPlM; however, the efficacy of each regimen cannot be compared due to the small sample size.

Three patients were treated with nivolumab, two of whom showed a long OS and second-line OS; however, the number of patients is too small to evaluate its efficacy (see Additional file 5). Recently, immunotherapy has shown promising results in patients with MPlM who progressed after at least one treatment line. In two phase II trials of MPlM [19, 20], nivolumab, an anti-programmed death-1 antibody, showed a median PFS of 2.6–6.1 months, and 6-month survival rates of 29–74%. Moreover, combination therapy with nivolumab and ipilimumab, an anti-cytotoxic T lymphocyte antigen 4 inhibitor, has shown an ORR of 28% and a median PFS of 5.6 months in a phase II trial of MPlM [21]. Although the efficacy of second-line chemotherapy with immune checkpoint inhibitors is not clear for MPeM, it is expected to improve survival considering the benefits for MPlM. A phase II trial of tremelimumab plus durvalumab for mesothelioma has shown an immune-related ORR of 28%; however, the clinical outcomes of MPeM are unknown [22]. A phase III trial of nivolumab in mesothelioma [23], including MPeM, is ongoing.

However, in this study, there was no remarkable difference in OS (time from first-line chemotherapy to death) between patients with and without subsequent chemotherapy after first-line failure. One explanation may be that patients who did not receive second-line chemotherapy included those who refused second-line chemotherapy, transferred hospitals, are under observation (which may include non-progressive disease), and are still undergoing first-line chemotherapy. These findings suggest that longer first-line chemotherapy may be recommended for patients in good general condition. Furthermore, it is necessary to select patients with MPeM who are suitable for second-line chemotherapy. In the univariate analysis to investigate the prognostic impact of age, sex, ECOG PS, histology, asbestos exposure, ascites, and distant organ metastasis, none of the covariates were significantly associated with second-line OS. The number of cycles of first-line platinum doublet chemotherapy showed a significant association with second-line OS and PFS. These findings suggest that second-line chemotherapy may be a good option and should be considered for patients in a good general condition who have completed 6 cycles of first-line chemotherapy. In our opinion, palliative care only, without any anticancer drugs, is an important option for patients with disease progression after < 6 cycles of first-line platinum-based chemotherapy, if they are in poor general condition or do not wish to continue chemotherapy.

There are some limitations to this study. First, this study was performed at a single institution, and the sample size was too small to accurately evaluate the efficacy of each regimen. Second, since this was a retrospective study and the patients’ background was not well-balanced, we could not compare the efficacy of each regimen. Third, the adverse event data collected retrospectively were insufficient. Finally, because of the characteristics of MPeM with few target lesions, we were unable to adequately assess the response.

## Conclusions

Second-line chemotherapy may be an option for patients with refractory MPeM, especially those who have completed 6 cycles of cisplatin or carboplatin plus pemetrexed as first-line chemotherapy.

## Supplementary Information

**Additional file 1.** Treatment response in patients with measurable lesions. CI, confidence interval; DCR, disease control rate (complete response, partial response, and stable disease).

**Additional file 2.** Univariate analysis of overall survival. CI, confidence interval; ECOG, Eastern Cooperative Oncology Group; HR, hazard ratio; NA, not assessed; OS, overall survival; PS, performance status.

**Additional file 3.** Kaplan–Meier curves of second-line overall survival according to the number of cycles of first-line chemotherapy. OS, overall survival.

**Additional file 4.** Kaplan–Meier curves of overall survival according to the number of treatment lines. OS, overall survival.

**Additional file 5.** Details of patients receiving nivolumab as second-line chemotherapy. ECOG, Eastern Cooperative Oncology Group; OS, overall survival; PFS, progression-free survival; PS, performance status.

## Data Availability

The datasets used and/or analysed during the current study are available from the corresponding author on reasonable request.

## Abbreviations

CI: Confidence interval
ECOG: Eastern Cooperative Oncology Group
MPeM: Malignant peritoneal mesothelioma
MPlM: Malignant pleural mesothelioma
NA: Not assessed
ORR: Overall response rate
OS: Overall survival
PFS: Progression-free survival
PS: Performance status
