# Supplementary material for: Efficacy of second-line treatment and prognostic factors in patients with advanced malignant peritoneal mesothelioma: a retrospective study
Source: BMC Cancer. 2021 Mar 20;21:294. doi: 10.1186/s12885-021-08025-x (PMC7980334; doi:10.1186/s12885-021-08025-x)
Supplement: Supplementary file 1 — Additional file 1. Treatment response in patients with measurable lesions. CI, confidence interval; DCR, disease control rate (complete response, partial response, and stable disease). [file 12885_2021_8025_MOESM1_ESM.docx]

**Additional file 1.** Treatment response in patients with measurable lesions

| Response | Patients (%) |
| --- | --- |
| First-line treatment | n = 25 (% in the first-line treatment) |
| Complete response | 0 (0.0) |
| Partial response | 5 (20.0) |
| Stable disease | 10 (40.0) |
| Progressive disease | 7 (28.0) |
| Not assessed | 3 (12.0) |
| Overall response rate | 5 (20.0), (95% CI: 6.8–40.7) |
| DCR | 15 (60.0), (95% CI: 38.7–78.9) |
| Second-line treatment | n = 12 (% in the second-line treatment) |
| Complete response | 0 (0.0) |
| Partial response | 1 (8.3) |
| Stable disease | 2 (16.7) |
| Progressive disease | 7 (58.3) |
| Not assessed | 2 (16.7) |
| Overall response rate | 1 (8.3) |
| DCR | 3 (25.0) |

Abbreviations: CI, confidence interval; DCR, disease control rate (complete response, partial response, and stable disease).
