# Supplementary material for: Efficacy of second-line treatment and prognostic factors in patients with advanced malignant peritoneal mesothelioma: a retrospective study
Source: BMC Cancer. 2021 Mar 20;21:294. doi: 10.1186/s12885-021-08025-x (PMC7980334; doi:10.1186/s12885-021-08025-x)
Supplement: Supplementary file 2 — Additional file 2. Univariate analysis of overall survival. CI, confidence interval; ECOG, Eastern Cooperative Oncology Group; HR, hazard ratio; NA, not assessed; OS, overall survival; PS, performance status. [file 12885_2021_8025_MOESM2_ESM.docx]

**Additional file 2.** Univariate analysis of overall survival

| Covariate |  | Median OS  (95% CI), months | HR  (95% CI) | p-value |
| --- | --- | --- | --- | --- |
| Age categorisation | < 70 | 15.4 (10.74–NA) | 1.00 (0.41–2.48) | 0.99 |
|  | ≥ 70 (reference) | 18.3 (6.74–NA) | – | – |
| Sex | Male | 15.4 (7.66–NA) | 1.85 (0.78–4.40) | 0.16 |
|  | Female (reference) | 16.9 (11.66–NA) | – | – |
| Ascites | Yes | 16.85 (12.25–NA) | 0.73 (0.29–1.81) | 0.49 |
|  | No (reference) | 7.59 (5.98–NA) | – | – |
| Asbestos exposure | Yes | 12.3 (6.74–NA) | 1.13 (0.51–2.49) | 0.77 |
|  | No or unknown (reference) | 16.9 (11.66–NA) | – | – |
| Histology | Epithelioid | 18.3 (11.66–NA) | 1.60 (0.76–3.36) | 0.20 |
|  | Others or unknown (reference) | 15.0 (7.59–NA) | – | – |
| ECOG PS | 0–1 | 16.56 (11.66–NA) | 2.03 (0.27–15.33) | 0.48 |
|  | 2 (reference) | 2.92 (2.92–NA) | – | – |
| Distant metastasis | Yes | 15.0 (7.59–NA) | 1.60 (0.77–3.33) | 0.2 |
|  | No (reference) | 16.9 (11.66–NA) | – | – |

Abbreviations: CI, confidence interval; ECOG, Eastern Cooperative Oncology Group; HR, hazard ratio; NA, not assessed; OS, overall survival; PS, performance status.
