# Supplementary material for: Efficacy of second-line treatment and prognostic factors in patients with advanced malignant peritoneal mesothelioma: a retrospective study
Source: BMC Cancer. 2021 Mar 20;21:294. doi: 10.1186/s12885-021-08025-x (PMC7980334; doi:10.1186/s12885-021-08025-x)
Supplement: Supplementary file 4 — Additional file 4. Kaplan–Meier curves of overall survival according to the number of treatment lines. OS, overall survival. [file 12885_2021_8025_MOESM4_ESM.pdf]

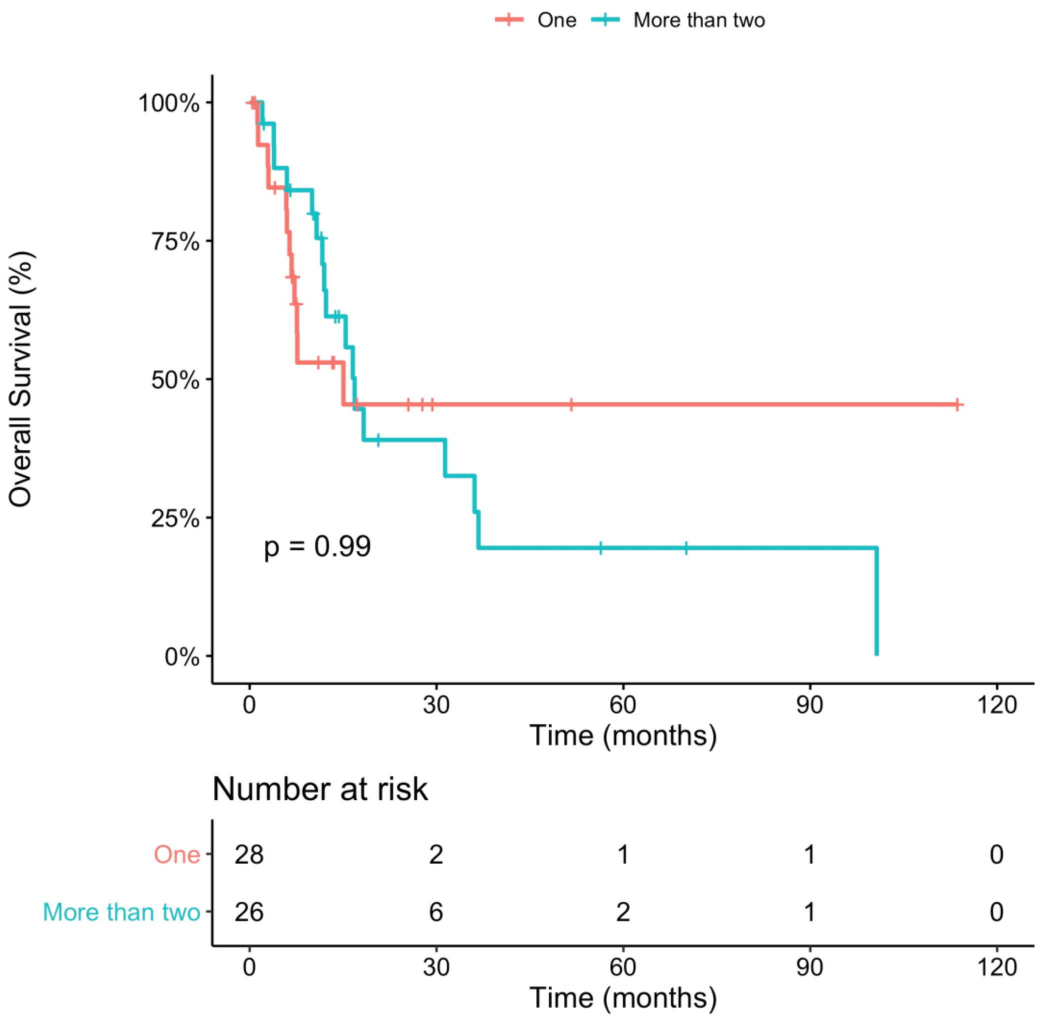


Additional file 4. Kaplan–Meier curves for OS according to the number of treatment lines. OS, overall survival.
