# Supplementary material for: Efficacy of second-line treatment and prognostic factors in patients with advanced malignant peritoneal mesothelioma: a retrospective study
Source: BMC Cancer. 2021 Mar 20;21:294. doi: 10.1186/s12885-021-08025-x (PMC7980334; doi:10.1186/s12885-021-08025-x)
Supplement: Supplementary file 5 — Additional file 5. Details of patients receiving nivolumab as second-line chemotherapy. ECOG, Eastern Cooperative Oncology Group; OS, overall survival; PFS, progression-free survival; PS, performance status. [file 12885_2021_8025_MOESM5_ESM.docx]

**Additional file 5.** Details of patients receiving nivolumab as second-line chemotherapy

| Case | Age | Histology | ECOG PS | Distant metastasis | Cycles of 1^st^ line | OS  (months) | PFS  (months) | 2^nd^ line OS  (months) |
| --- | --- | --- | --- | --- | --- | --- | --- | --- |
| 1 | 60 | Epithelioid | 0 | Yes | 6 | 36.7 | 8.0 | 12.6 |
| 2 | 74 | Sarcomatoid | 1 | Yes | 6 | 20.7 | 12.6 | 12.6 |
| 3 | 51 | Mixed | 1 | Yes | 2 | 2.0 | 0.2 | 0.7 |

Abbreviations: ECOG, Eastern Cooperative Oncology Group; OS, overall survival; PFS, progression-free survival; PS, performance status.
